# Supplementary material for: Transoral Robotic Surgery for Oropharyngeal Squamous Cell Carcinoma of the Tonsil versus Base of Tongue: A Systematic Review and Meta-Analysis
Source: Cancers (Basel). 2022 Aug 8;14(15):3837. doi: 10.3390/cancers14153837 (PMC9367622; doi:10.3390/cancers14153837)
Supplement: Supplementary file 1 [file cancers-14-03837-s001.zip › cancers-1856711-supplementary.pdf]

Supplementary Material: Transoral Robotic Surgery for Oropharyngeal Squamous Cell Carcinoma of the Tonsil versus Base of Tongue: A Systematic Review and Meta-Analysis

Nicolas S Poupore, Tiffany Chen, Shaun A Nguyen, Cherie-Ann O Nathan and Jason G Newman

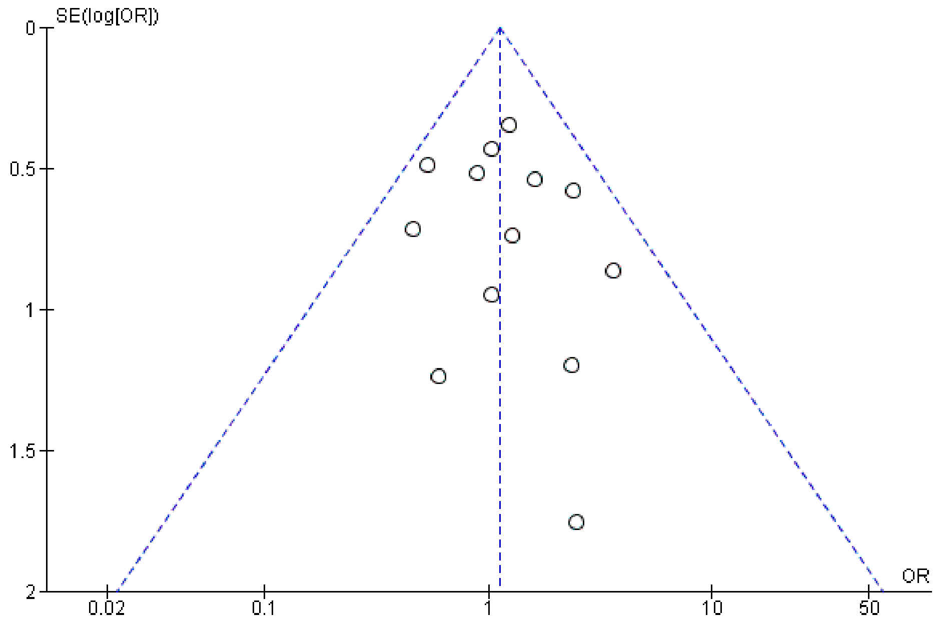

Figure S1. Funnel plot assessing for publication bias.
